# CT-Based Morphometric Analysis of Zygomaticomaxillary Suture Symmetry: Implications for Diagnostic Imaging

**DOI:** 10.3390/diagnostics15182330

**Published:** 2025-09-15

**Authors:** Atakan Kırteke, Hilal Gören, Nilgün Tuncel Çini

**Affiliations:** 1 Department of Radiology, Nev Hospital, Sanlıurfa 63300, Turkey; atakankirteke@gmail.com; 2 Department of Anatomy, Faculty of Medicine, Bilecik Şeyh Edebali University, Bilecik 11230, Turkey; hilal.goren@bilecik.edu.tr

**Keywords:** zygomaticomaxillary suture, craniofacial symmetry, mirror-based modeling, morphometric analysis, regression modeling

## Abstract

**Background/Objectives**: Bilateral symmetry of craniofacial structures is a fundamental principle in clinical application, particularly in procedures involving unilateral trauma or skeletal loss. The zygomaticomaxillary suture (ZMS), located at the articulation between the zygomatic bone and maxilla, is considered a potentially stable midfacial landmark owing to its distinct anatomical position and relevance in surgical planning. This study aimed to evaluate the bilateral symmetry of the ZMS and its surrounding anatomical structures in healthy adults using three-dimensional CT reconstructions and to develop predictive models for contralateral estimation. **Methods**: Craniofacial CT scans of 200 adult individuals (101 females and 99 males, aged ≥18 years) were retrospectively analyzed. Fourteen morphometric parameters related to the ZMS and adjacent craniofacial structures were measured bilaterally on 3D reconstructions generated from CT data. Statistical analyses included tests for normality, sex and side comparisons, correlation analysis, and linear regression to develop side-predictive formulas. **Results**: No statistically significant differences were found between the right and left sides for any parameter, confirming a high degree of bilateral symmetry. However, significant sex-based differences were observed in two parameters: the lateral extension of the ZMS (*p* = 0.024 right; *p* = 0.046 left) and piriform aperture width (*p* = 0.017). Regression models developed for each sex provided reliable estimates of contralateral morphometric values based on single-sided measurements. **Conclusions**: The results confirm high bilateral symmetry of the ZMS and adjacent midfacial structures, supporting its reliability as a reference point in surgical planning and facial reconstruction. Regression models enhance the accuracy of mirror-based approaches in unilateral midfacial defects.

## 1. Introduction

Craniofacial symmetry is a fundamental determinant of both aesthetic appearance and functional harmony [1]. Accurate knowledge of skeletal symmetry is not only essential for maxillofacial surgery and prosthetic design but also plays a pivotal role in diagnostic imaging, trauma management [2]. Among the key anatomical landmarks in the midfacial region is the zygomaticomaxillary suture (ZMS), which forms the lateral boundary of the orbital floor by articulating the zygomatic bone with the maxilla [3]. Despite its clinical importance in surgical planning and fracture management, the contribution of the ZMS to overall facial symmetry has received limited attention in the literature. Existing studies predominantly focus on larger craniofacial structures such as the orbits, nasal bones, and mandible, while craniofacial sutures remain largely underexplored in morphometric symmetry evaluations [4,5,6].

Owing to its distinct anatomical location, the ZMS has been reported to exhibit consistent visibility on computed tomography (CT) imaging, which highlights its potential utility as a reference structure in clinical surgical planning and reconstructive procedures [7]. Postoperative imaging studies have reported that the ZMS can achieve near-symmetric alignment following surgical intervention, reinforcing its utility in midfacial reconstruction [6,8]. However, most of these findings are based on clinical populations with underlying trauma or pathology. As such, they may not fully reflect the normative anatomical symmetry observed in healthy individuals. This gap in the literature highlights the need for morphometric investigations targeting healthy samples to objectively evaluate bilateral ZMS symmetry, especially in cases where mirror-based reconstruction strategies are applied due to unilateral skeletal loss.

Facial reconstruction, whether undertaken in the context of trauma, oncologic resection, or congenital deformity, is highly dependent on the precise restoration of midfacial symmetry [9,10]. These procedures assume craniofacial symmetry, allowing estimation of missing or damaged anatomical structures from the contralateral side [11]. Thus, the identification of stable and reproducible reference points is essential. Sutural landmarks such as the ZMS, with their consistent anatomical integrity, provide surgeons with dependable guidance, enhancing the precision of mirror-based reconstructions and ultimately improving clinical outcomes.

Despite the increasing reliance on mirror-based modeling in surgical reconstruction, there is a lack of quantitative data validating the bilateral symmetry of midfacial sutures. Given its strategic position at the articulation between the zygomatic bone and maxilla, the ZMS may serve as a reliable anatomical landmark when conventional midfacial reference points are damaged or unavailable [6,8].

Therefore, the present study aims to quantitatively assess the bilateral symmetry of the ZMS and adjacent craniofacial landmarks using CT data from healthy individuals. In contrast to the majority of previous symmetry studies that concentrated on larger craniofacial structures, the present study highlights a sutural landmark, thereby drawing attention to an underexplored dimension of craniofacial symmetry. Through detailed morphometric analysis and evaluation of side-to-side and sex-based differences, this research seeks to provide anatomically robust reference data to inform midfacial surgical planning. Additionally, regression models were developed to estimate contralateral morphometric values, offering a practical approach to unilateral reconstruction and supporting the anatomical validity of mirror-based modeling techniques.

## 2. Materials and Methods

### 2.1. Study Design and Sample

This retrospective study received ethical approval from the Clinical Research Ethics Committee of Harran University (Approval No: HRÜ/25.12.40, Meeting No: 12, Date: 30 June 2025) and was carried out in compliance with the ethical standards of the Declaration of Helsinki.

Craniofacial CT scans of 200 adult individuals (101 females and 99 males; aged between 18 and 40 years) were retrospectively evaluated. The mean age was 29.25 years for females and 29.81 years for males. All CT scans were obtained as part of routine clinical practice (e.g., paranasal sinus evaluation, assessment of chronic headache). To ensure a representative healthy study population, individuals with a history of craniofacial trauma, pathological conditions, congenital anomalies, or previous surgical interventions were excluded. The inclusion criteria were full visibility of the ZMS and adjacent midfacial structures, while CT images with artifacts or asymmetries affecting the midface were excluded.

### 2.2. Image Acquisition Protocol

All CT scans were acquired using a GE Revolution Maxima scanner (GE Hangwei Medical Systems Co., Ltd., Beijing, China). The imaging parameters were set at 100 kV and 348 mA, with a slice thickness of 1.25 mm. Axial images were obtained at 2 mm intervals and reconstructed using a 512 × 512 matrix.

The maxillofacial region was imaged in high resolution, and the thin-section (1.25 mm) axial images were transferred to a dedicated workstation (AW VolumeShare 7; GE HealthCare, Beijing, China) for post-processing. Three-dimensional reconstructions were generated, and all morphometric measurements were performed on these 3D models to ensure spatial accuracy and anatomical consistency.

### 2.3. Morphometric Parameters

**Figure 1:**
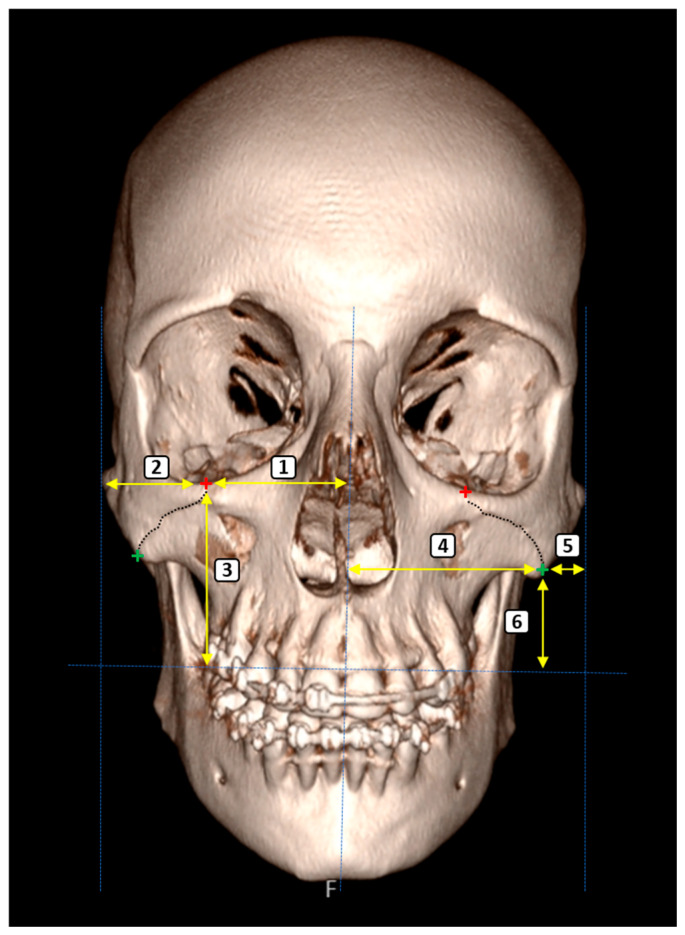
Three-dimensional anterior view of the craniofacial skeleton illustrating bilateral morphometric evaluation of the ZMS and its anatomical relationships. Red and green crosses mark the superior and inferior articulation points of the ZMS on the infraorbital margin and lateral zygomatic border, respectively. The dashed lines represent the plans and the yellow lines represent the parameters. The numbered parameters correspond to: (1) Distance from the superior ZMS to the midsagittal plane; (2) Distance between the superior ZMS and the lateral zygomatic border; (3) Vertical distance from the superior ZMS to the plane passing through the supradentale; (4) Distance from the inferior ZMS to the midsagittal plane; (5) Distance between the inferior ZMS and the lateral zygomatic border; (6) Vertical distance from the inferior ZMS to the plane passing through the supradentale.

**Figure 2:**
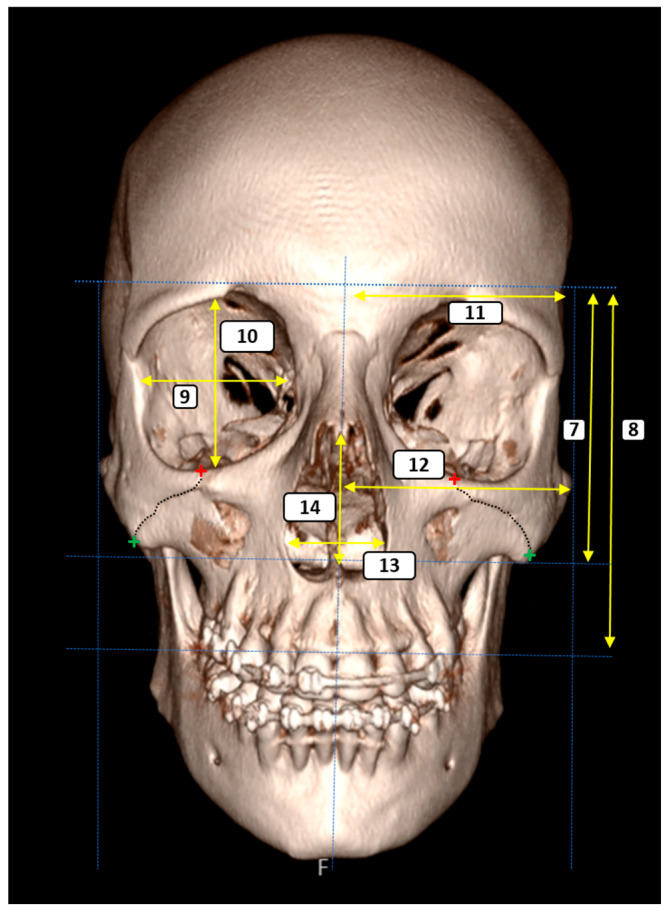
Three-dimensional anterior view of the craniofacial skeleton showing morphometric assessment of the ZMS and selected craniofacial landmarks. Red and green crosses indicate the superior and inferior articulation points of the ZMS, respectively. The dashed lines represent the plans and the yellow lines represent the parameters. The numbered measurements represent: (7, 8) vertical facial proportions from the glabella to the inferior zygomatic and supradentale planes; (9, 10) orbital width and height; (11, 12) transverse distances from eurion and zygion to the midsagittal plane; (13, 14) width and height of the piriform aperture.

**Figure 3:**
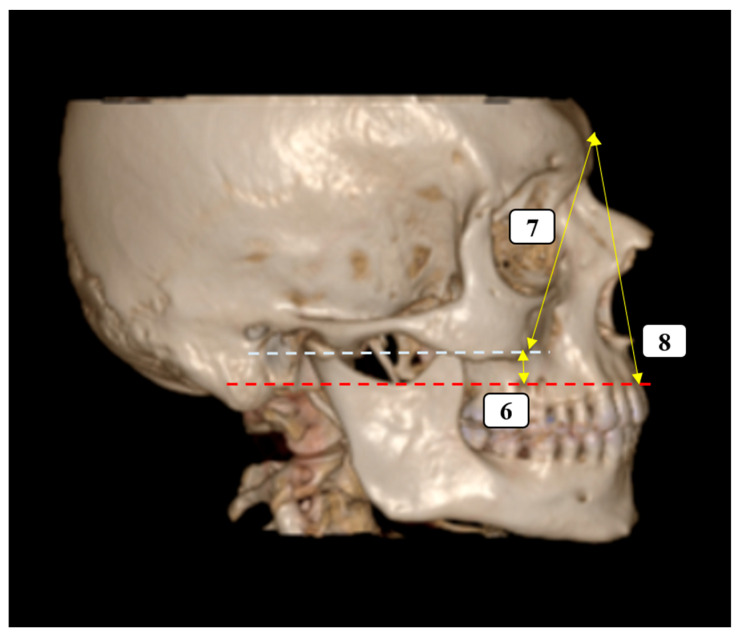
Parameters measured from different angles (Lateral view). The dashed lines represent the plans and the yellow lines represent the parameters. (6) Vertical distance from the inferior ZMS to the plane passing through the supradentale; (7, 8) vertical facial proportions from the glabella to the inferior zygomatic and supradentale planes.

A total of 14 morphometric parameters were selected to quantitatively assess craniofacial symmetry and the anatomical relationships surrounding the ZMS. The measurements of the parameters used in the study were carried out by the radiologist (A.K.) at one-week intervals, and mean values were used in the statistical analyses. All reference points were clearly identifiable on the 3D reconstructed CT images, and measurements were performed bilaterally unless otherwise specified. The parameters were grouped according to anatomical region and functional relevance as follows (Figure 1, Figure 2 and Figure 3):

- ZMS-Related Measurements

These parameters were designed to evaluate the spatial position of the ZMS with respect to surrounding craniofacial landmarks:

1. ZMS–Midline Distance (Superior): The horizontal distance from the superimargin of the ZMS at the infraorbital rim to the midsagittal plane.
2. ZMS–Lateral Zygomatic Border (Superior): The distance between the superior ZMS and the lateral edge of the zygomatic bone.
3. ZMS–Supradentale Plane (Superior): The vertical distance from the superior ZMS to the horizontal plane passing through the supradentale.
4. ZMS–Midline Distance (Inferior): The horizontal distance from the inferior margin of the ZMS, located along the inferior zygomatic border, to the midsagittal plane.
5. ZMS–Lateral Zygomatic Border (Inferior): The distance from the inferior ZMS to the lateral zygomatic border.
6. ZMS–Supradentale Plane (Inferior): The vertical distance between the inferior ZMS and the horizontal plane aligned with the supradentale.

- Vertical Facial Proportions

To assess vertical facial harmony and height relationships, the following parameters were recorded:

7. Glabella–Inferior Zygomatic Plane Distance: The vertical distance between the horizontal planes passing through the glabella and the inferior zygomatic border.
8. Glabella–Supradentale Plane Distance: The vertical distance between the glabella and supradentale reference planes.

- Orbital Morphometry

Standard orbital dimensions were measured to examine orbital symmetry and shape:

9. Orbital Width: The maximum horizontal diameter of the orbit.
10. Orbital Height: The maximum vertical diameter of the orbit.

- Transverse Craniofacial Dimensions

These parameters were used to evaluate facial and cranial width with respect to the midsagittal axis:

11. Eurion–Midline Distance (bilateral): The distance from the eurion point (maximum cranial breadth) to the midsagittal plane.
12. Zygion–Midline Distance (bilateral): The distance from the zygion point (maximum facial breadth) to the midsagittal plane.

- Piriform Aperture Morphometry

To assess the nasal cavity morphology, the following dimensions were included:

13. Piriform Aperture Width: The distance between the alare points.
14. Piriform Aperture Height: The vertical distance between the nasospinale and rhinion.

### 2.4. Statistical Analysis

Morphometric data obtained in the study were analyzed using SPSS 25.0 (IBM Corp., Armonk, NY, USA) statistical software. Measurement data were evaluated with descriptive statistics, mean ± standard deviation for parametric variables and median (interquartile range) for non-parametric variables, and also minimum and maximum values were given for each parameter.

The normality of data distribution was evaluated with the Shapiro–Wilk test. Since the data did not show a normal distribution, the Mann–Whitney *U* test was used for the side and sex comparisons (*p* < 0.05). Spearman correlation analyses were applied to determine the relationship levels between parameters (*p* < 0.01). Formulas were produced for females and males to predict the data of the other side using one side or vice versa, with one-way linear regression analysis.

## 3. Results

Descriptive statistical values of the parameters measured in male and female individuals were given in Table 1. Accordingly, no difference was observed between the right and left sides for any parameter. Although there was no difference between the parties, significant differences were observed between males and females for two parameters. It was observed that there was a sex difference in the distance between the starting point of the ZMS on the lower orbital margin and the lateral margin of the zygomatic bone (*p* = 0.024 for the right, *p* = 0.046 for the left side), and the width of the piriform aperture (*p* = 0.017).

According to the correlation analysis performed by side, no correlation value that could be considered significant between the parameters was detected. Since the data obtained did not show any difference between the sides, it was assumed that the bone structures were symmetrical. Formulas were developed to predict the other side using data from one side, based on this structural feature. Since the two parameters show sex differences, the formulas were produced separately for females and males. Accordingly, the formulas that will estimate the left side using the data from the right side or vice versa using the data from the left side were given in Table 2 and Table 3.

## 4. Discussion

The present study aimed to systematically evaluate the bilateral symmetry of the ZMS and its spatial relationship with adjacent craniofacial landmarks using retrospective computed tomography imaging in a healthy adult population. The findings demonstrated a high degree of side-to-side symmetry in all measured parameters, supporting the anatomical validity of the ZMS as a reliable midfacial reference point. Notable sex-based differences were observed in select measurements, particularly in the lateral extension of the ZMS and the width of the piriform aperture, indicating the influence of sexual dimorphism on midfacial architecture. While previous studies have typically examined craniofacial symmetry through broader structures such as the orbit or mandible, comprehensive morphometric analyses of midfacial sutures in healthy samples stratified by sex remain limited [12,13,14]. By integrating sex-based comparisons with precise skeletal measurements, the present study offers a refined understanding of anatomical variability in the midface. These results underscore the importance of the ZMS as a reliable landmark in surgical practice, particularly in cases of unilateral trauma or skeletal loss, where contralateral reference–based modeling may improve the precision of reconstruction procedures.

The high degree of bilateral symmetry observed in the present study is consistent with previous research emphasizing craniofacial symmetry as a foundational concept in surgical planning [11,15]. Studies utilizing automatic three-dimensional facial surface analyses have similarly reported strong bilateral correspondence in midfacial regions, including the zygomatic and maxillary areas, supporting the assumption that these structures exhibit consistent morphological patterns across sides [6,16,17].

Our findings further support this anatomical consistency by providing morphometric evidence from skeletal landmarks directly related to the ZMS. Given the ZMS’s strategic location at the intersection of key midfacial bones, its symmetrical positioning with adjacent structures such as the orbit, supradentale, and piriform aperture strengthens its role as a stable and reproducible reference point. This interpretation is further supported by previous shape-based analyses, which have reported that the ZMS contour demonstrates a notable degree of bilateral similarity within individuals, suggesting a generally symmetrical morphological pattern [3].

In clinical contexts, particularly in midfacial reconstructive surgery approximation, the use of contralateral ZMS data offers a reliable anatomical guide when unilateral structures are compromised due to trauma or pathology [18,19]. Indeed, our data demonstrate that ZMS and adjacent skeletal landmarks display high bilateral concordance—within millimetric thresholds—indicating sufficient positional stability to support mirroring-based surgical modeling. This interpretation is in line with published three-dimensional assessments of midfacial symmetry, where comparisons between true and contralaterally mirrored models of the midfacial complex revealed mean deviations under 1 mm, validating the clinical utility of a mirrored reference framework [16]. This underscores the practical utility of incorporating such morphometric landmarks into individualized modeling protocols for enhanced procedural accuracy.

Sex-based differences observed in the lateral extension of the ZMS and the transverse width of the piriform aperture are consistent with established findings on craniofacial sexual dimorphism. In a CT-based morphometric study, Moreddu et al. (2013) reported that males exhibited significantly greater piriform aperture widths (25.32 mm) compared to females (24.00 mm), along with increased vertical heights (36.35 mm in males vs. 32.54 mm in females) [20]. Similar patterns have been identified by Sarač-Hadžihalilović et al. (2022), who demonstrated distinct sex-related differences in both the size and shape of the piriform aperture using geometric morphometric techniques [21]. Similarly, previous research has reported that males tend to exhibit larger midfacial skeletal dimensions, with size emerging as a more decisive factor than shape in sex determination [22].

In line with these observations, our study revealed statistically significant sex-related differences in both ZMS extension and piriform aperture width. Specifically, the distance between the ZMS origin at the infraorbital margin and the lateral border of the zygomatic bone was significantly greater in males than in females on both sides (*p* = 0.024 right; *p* = 0.046 left), and the piriform aperture was also wider in males (*p* = 0.017), reflecting sex-related differences in midfacial morphology. These findings not only reinforce the role of ZMS-associated landmarks in capturing sex-based skeletal variability, but also underscore their clinical and in procedures that rely on accurate morphological differentiation, such as sex estimation and individualized facial reconstruction.

Traditional symmetry assessments have predominantly focused on broader craniofacial regions; however, such approaches may fall short in detecting finer anatomical asymmetries [23,24,25,26]. In contrast, targeted analyses centered on the ZMS and its surrounding structures allow for higher-resolution evaluation of bilateral congruence, thereby enhancing anatomical precision and procedural accuracy in symmetry-based reconstruction protocols. Consistently, our study offers detailed morphometric insight into the ZMS and its neighboring landmarks, revealing a high degree of bilateral symmetry with only minimal differences between sides, and reinforcing the value of using such targeted reference points in clinical applications.

Facial reconstruction, using the intact side of the face as a guide has become a widely accepted approach, particularly in cases involving unilateral trauma or bone loss [10]. Mirroring techniques—where the unaffected side is digitally duplicated—hold significant potential for accurately restoring midfacial symmetry. Several studies have shown that deviations associated with this method are generally minimal, supporting its anatomical reliability [27,28,29]. As the primary component of the maxillofacial region, the ZMS has the potential to be a reliable reference point for facial reconstruction in the presence of such conditions. Our findings further support this approach, revealing a notable degree of symmetry between the ZMS and its adjacent skeletal landmarks, with only minor differences observed between sides. This suggests that the ZMS is not only a stable and well-defined skeletal structure, but also a dependable reference point in cases where precision is essential for reconstruction. Owing to its central location and consistent alignment with neighboring structures such as the orbit and piriform aperture, the ZMS provides a robust anatomical anchor for symmetry-based modeling. These results reinforce the clinical of using the contralateral side as a template, contributing to the reliable and anatomically accurate restoration of facial form.

The pronounced symmetry of the ZMS can be explained by its embryological origin from neural crest–derived elements, which develop in a bilaterally coordinated fashion [30]. Moreover, its position at the junction of the zygomatic bone and maxilla, both exposed to symmetrical masticatory loading, likely reinforces structural balance. In contrast to larger craniofacial structures that are more susceptible to growth-related variability and environmental influences, sutural landmarks such as the ZMS exhibit greater developmental stability, underscoring their reliability as reference points in clinical practice.

Regression analysis in this study demonstrated that the position of the ZMS on one side of the face can be reliably predicted using linear measurements from the opposite side. Most of the models showed strong statistical performance, with adjusted R^2^ values frequently exceeding 0.90 and low standard errors, indicating high anatomical consistency and predictive accuracy. These findings suggest that, in cases of unilateral skeletal loss or trauma, the intact side can serve as a reliable reference for estimating midfacial anatomy. Additionally, the use of separate regression models for male and female individuals may enhance the precision and individualization of applications such as facial reconstruction approximation. It’s important to emphasize that among these formulas, the adjusted R^2^ values, and therefore the percentage of accurate predictions, are very low for the ZMS-superior midline and ZMS-inferior midline parameters, particularly in males. While the sexual dimorphism of the ZMS-inferior midline parameter supports this data, we also note that it represents a much less reliable distance in terms of symmetry than the other parameters.

## 5. Conclusions

This study comprehensively evaluated the morphometric characteristics of the ZMS and surrounding midfacial structures using CT images from an adult sample. The findings demonstrated a high degree of right–left symmetry across all measurements, confirming the reliability of the ZMS as an anatomical reference point. Although minor sex-related differences were observed in certain parameters, they did not affect the overall symmetry. The regression models developed offer a practical solution for estimating contralateral measurements, especially in cases of unilateral skeletal loss. These results indicate that the ZMS can serve as an effective reference structure for individualized anatomical mapping in applications such as surgical planning and facial reconstruction. As a future direction, it would also be valuable to investigate whether cranial asymmetry displays sex-specific traits, thereby providing predictive potential for sex estimation.

## 6. Limitations

This study has certain limitations. First, the sample consisted exclusively of adult individuals without craniofacial anomalies, which may limit the generalizability of the findings to pediatric populations or those with specific clinical conditions. Furthermore, although the regression models provide practical estimations in unilateral cases, their accuracy should be further validated in individuals with asymmetric morphology or a history of trauma. In addition, potential measurement errors should be acknowledged, as no formal calibration procedure or assessment of intra-observer variability was performed. Finally, the study was conducted on a single population, which may restrict the broader applicability of the results across diverse demographic groups.

## Figures and Tables

**Table 1:** Descriptive and comparative statistical values for right and left sides for females and males (in mm).

	Female (*n* = 101)					Male (*n* = 99)				*p* Value Within Sex		
	Right		Left		*p* Value	Right		Left		*p* Value	Right	Left
**1**	33.00 (5.00)	25.00–47.00	34.00 (4.50)	25.00–46.00	0.496	38.00 (5.00)	25.00–40.00	33.00 (5.00)	25.00–43.00	0.657	0.954	0.956
**2**	32.0 (6.00)	23.00–52.00	32.00 (5.00)	24.00–56.00	0.892	33.00 (4.00)	21.00–53.00	33.00 (5.00)	20.00–53.00	0.749	**0.024**	**0.046**
**3**	45.43 ± 6.41	31.00–58.00	45.37 ± 6.10	32.00–59.00	0.946	45.97 ± 5.98	33.00–59.00	45.97 ± 5.99	29.00–59.00	0.895	0.536	0.401
**4**	48.33 ± 7.48	31.00–67.00	45.82 ± 7.50	32.00–65.00	0.851	47.84 ± 7.53	31.00–67.00	47.89 ± 7.55	31.00–69.00	0.962	0.646	0.551
**5**	9.00 (3.00)	5.00–14.00	9.00 (4.00)	4.00–16.00	0.805	9.00 (3.00)	5.00–19.00	9.00 (3.00)	4.00–19.00	0.361	0.287	0.202
**6**	28.00 (7.00)	19.00–45.00	28.00 (8.00)	20.00–47.00	0.998	29.00 (8.00)	20.00–50.00	30.00 (7.00)	20.00–50.00	0.971	0.428	0.491
**7**	59.00 (16.50)	41.00–90.00	59.00 (16.50)	42.00–91.00	0.854	59.00 (11.00)	33.00–87.00	59.00 (9.00)	29.00–89.00	0.592	0.719	0.824
**8**	79.00 (13.00)	45.00–92.00	80.00 (13.50)	45.00–91.00	0.410	80.00 (15.00)	49.00–95.00	81.00 (14.00)	48.00–95.00	0.533	0.194	0.122
**9**	42.00 (10.00)	31.00–70.00	42.00 (9.50)	33.00–72.00	0.500	42.00 (6.00)	36.00–65.00	42.00 (6.00)	6.00–65.00	0.883	0.428	0.683
**10**	43.00 (18.00)	26.00–77.00	44.00 (16.00)	29.00–79.00	0.757	43.00 (12.00)	27.00–71.00	45.00 (13.00)	27.00–72.00	0.717	0.577	0.475
**11**	55.00 (8.00)	39.00–70.00	55.00 (7.50)	39.00–72.00	0.839	55.00 (8.00)	31.00–72.00	56.00 (7.00)	31.00–74.00	0.558	0.994	0.709
**12**	52.54 ± 5.62	40.00–65.00	52.57 ± 5.93	39.00–69.00	0.971	52.57 ± 6.49	38.00–67.00	52.56 ± 6.30	36.00–67.00	0.981	0.768	0.759
**13**	25.00 (3.50)		20.00–29.00		-	26.00 (3.00)		21.00–29.00		-	0.017	
**14**	36.00 (3.00)		29.00–40.00		-	36.00 (3.00)		30.00–39.00		-	0.998	

The bold *p* value indicates the parameter that shows a significant difference.

**Table 2:** Regression formulas for females.

Female					
Formulas	Adjusted R^2^	SE	Formulas	Adjusted R^2^	SE
Right 1 = 4.21 + (0.862 × Left 1)	0.821	1.591	Left 1 = 1.93 + (0.954 × Right 1)	0.821	1.674
Right 2 = 2.11 + (0.933 × Left 2)	0.930	1.694	Left 2 = 0.24 + (0.998 × Right 2)	0.930	1.752
Right 3 = −0.499 + (1.01 × Left 3)	0.927	1.732	Left 3 = 3.37 + (0.916 × Right 3)	0.927	1.648
Right 4 = 1.23 + (0.97 × Left 4)	0.946	1.738	Left 4 = 1.38 + (0.975 × Right 4)	0.946	1.742
Right 5 = 3.07 + (0.638 × Left 5)	0.605	1.442	Left 5 = 0.62 + (0.954 × Right 5)	0.605	1.764
Right 6 = 1.77 + (0.935 × Left 6)	0.931	1.533	Left 6 = 0.24 + (0.996 × Right 6)	0.931	1.582
Right 7 = 1.54 + (0.97 × Left 7)	0.981	1.604	Left 7 = −0.351 + (1.011 × Right 7)	0.981	1.638
Right 8 = 0.34 + (0.988 × Left 8)	0.976	1.666	Left 8 = 1.49 + (0.988 × Right 8)	0.976	1.666
Right 9 = −2.02 + (1.038 × Left 9)	0.961	1.544	Left 9 = 3.59 + (0.926 × Right 9)	0.961	1.459
Right 10 = −0.199 + (0.996 × Left 10)	0.964	1.809	Left 10 = 1.85 + (0.969 × Right 10)	0.964	1.785
Right 11 = 5.36 + (0.896 × Left 11)	0.803	2.642	Left 11 = 5.75 + (0.898 × Right 11)	0.803	2.644
Right 12 = 5.75 + (0.890 × Left 12)	0.881	1.939	Left 12 = 0.50 + (0.991 × Right 12)	0.881	2.046

SE; Standard Estimation.

**Table 3:** Regression formulas for males.

Male					
Formulas	Adjusted R^2^	SE	Formulas	Adjusted R^2^	SE
Right 1 = 35.57 + (−0.098 × Left 1)	0.039	3.304	Left 1 = 28.44 + (0.109 × Right 1)	0.037	3.443
Right 2 = 65.65 + (−0.876 × Left 2)	0.084	5.418	Left 2 = 25.10 + (0.318 × Right 2)	0.091	5.371
Right 3 = 1.11 + (0.973 × Left 3)	0.921	1.678	Left 3 = 2.53 + (0.947 × Right 3)	0.921	1.656
Right 4 = 1.42 + (0.969 × Left 4)	0.942	1.814	Left 4 = 1.36 + (0.972 × Right 4)	0.942	1.817
Right 5 = 2.72 + (0.680 × Left 5)	0.648	1.404	Left 5 = 0.74 + (0.958 × Right 5)	0.648	1.667
Right 6 = 1.79 + (0.937 × Left 6)	0.930	1.489	Left 6 = 0.30 + (0.993 × Right 6)	0.930	1.533
Right 7 = 3.07 + (0.944 × Left 7)	0.976	1.640	Left 7 = −1.67 + (1.034 × Right 7)	0.976	1.716
Right 8 = 0.533 + (0.984 × Left 8)	0.977	1.666	Left 8 = 1.23 + (0.993 × Right 8)	0.977	1.674
Right 9 = 10.58 + (0.765 × Left 9)	0.734	3.592	Left 9 = 1.46 + (0.963 × Right 9)	0.734	4.032
Right 10 = 1.18 + (0.946 × Left 10)	0.958	1.766	Left 10 = 0.72 + (0.995 × Right 10)	0.958	1.794
Right 11 = 2.62 + (0.943 × Left 11)	0.933	1.561	Left 11 = 1.03 + (0.99 × Right 11)	0.935	1.574
Right 12 = 0.50 + (0.991 × Left 12)	0.923	1.795	Left 12 = 3.51 + (0.933 × Right 12)	0.923	1.742

SE; Standard Estimation.

## Data Availability

The data presented in this study are available on request from the corresponding author. The data are not publicly available due to privacy.

## Abbreviations

The following abbreviations are used in this manuscript:

ZMS	Zygomaticomaxillary suture
CT	Computed Tomography
